# Editorial: When Physics Meets Biology; Biomechanics and Biology of Traumatic Brain Injury

**DOI:** 10.3389/fneur.2016.00091

**Published:** 2016-06-15

**Authors:** Denes V. Agoston, Mattias K. Sköld

**Affiliations:** ^1^Department of Anatomy, Physiology and Genetics, Uniformed Services University, Bethesda, MD, USA; ^2^Department of Neuroscience, Karolinska Institutet, Stockholm, Sweden; ^3^Department of Neurosurgery, Uppsala University, Uppsala, Sweden

**Keywords:** physics, forces, biology, response, brain

**The Editorial on the Research Topic**

**When Physics Meets Biology; Biomechanics and Biology of Traumatic Brain Injury**

We have launched this special topic to focus on the critical relationships between physical forces, biomechanics, and biological responses in traumatic brain injury (TBI). Understanding the precise connection between physical force(s) and biological response(s), the “physical to biological coupling” is essential for developing high fidelity models in experimental TBI, for designing better protective devices and measures, for establishing more accurate diagnostics, and – most importantly – for identifying specific, evidence-based pharmacotherapies for TBI.

Several of the contributions are dedicated to the very important topic of modeling various forms of brain trauma describing an *in vitro* model of cavitation (Cao et al.), models of mild TBI (Chen et al.). Blast-induced TBI poses special challenge for modeling and two reviews are dedicated to address some of the issues associated with blast-induced TBI research (Courtney and Courtney; Needham et al.). As US epidemiology data show, the importance of understanding penetrating TBI – sadly – has became even more urgent. Cernak et al. describe a novel model for penetrating TBI, whereas Davidsson and Risling provide a great example of using finite element modeling in penetrating TBI. This study, together with a review by Carlsen and Daphalapurkar, focusing on the importance of structural anisotropy in biofidelity of computational models, both include the possibility for more sophisticated material definitions and can implement increasingly more physiologically relevant measures of injury. The overview by Young et al. fills a critical gap by comparing the physics of high velocity penetrating, blunt impact and blast injuries.

As these contributions illustrate, modeling TBI is a truly multidimensional problem. The first dimension, the physical forces are highly variable and complex. They vary from relatively low-speed, low kinetic energy, typical in traffic and sport accidents to high velocity, high kinetic energy type observed in explosive blast. The next dimension is how these forces interact with the head and ultimately with the brain, whether they interact with the entire head during acceleration–deceleration causing diffuse, closed head TBI, or if they are high velocity, concentrated kinetic energy propelling, e.g., a bullet penetrating the skull and brain parenchyma causing focal, open TBI, or the combination of various velocities and intensities. The next important, yet currently, understudied dimension in modeling is to take into account the directionality of the physical forces relative to the anatomy of the brain. The use of the new generation of sensors, such as the Vector mouth guard (i1 Biometrics, Inc., Kirkland, WA, USA) or the Prevent™ Concussion Impact Monitor Mouthguard (Prevent Biometrics, MN, USA) in combination with functional and molecular outcome measures can provide data not only about the g-forces but also about vectorial information in human/clinical TBI. Precise readout of g-forces and their directionality relative to neuroanatomical structures coupled with neurobehavioral and molecular outcome measures has the potential to elevate current sensor technology to a whole new level. While the biological responses to the various physical forces are quite similar, there are pathological changes of that are predominantly triggered by specific physical force. Such example is axonal injury in response to rotational forces. Most pathobiologies of the secondary injury process, metabolic changes, vascular damage, inflammation, etc., seem to occur independent of the physical force or directionality. However, the sequelae, the onset, and extent of the individual pathologies appear to be affected by the causative physical force. It is, thus, conceivable that future sensors using “big data” approaches will be able to provide predictive information about the biological response to the physical impact. Such system will be able to identify individuals with increased cerebral vulnerability and will guide safe return to play or duty. Similarly, such advanced sensor system will be able to recommend personalized treatments taking into account the injured individual’s biology, medical history, etc. Unfortunately, no similar sensors are currently in use in experimental TBI. This further increases the gap between experimental and clinical TBI studies; in clinical TBI, we can determine the extent of functional deficits and using the new generation of sensors, we can increasingly measure the causative physical forces. In experimental TBI, on the other hand, we set the *indirect* parameters of the injury (e.g., pressure in fluid percussion) but we do not measure the head movement (where applicable) and only infrequently the type and extent of the functional deficits caused by the indirect physical forces.

Our hope is that the new knowledge derived from the better understanding of “physical to biological coupling” will also lead to improved helmet design, car safety, etc. We also hope that the ability to connect the physics to biological outcomes will help to classify TBI cases based on the physical forces, directionality, and biological responses, rather than the currently used Glasgow Coma scale (GCS). The failure to develop specific and effective pharmacotherapies in TBI, despite of the many promising preclinical studies, may partly stem from lack of understanding and modeling of the very physical forces that trigger the specific secondary process.

We hope that this research topic will contribute toward closing the gap between experimental and clinical TBI studies. We believe that such a “back to basics” approach is much needed and warranted and will help researchers, and the many new scientists entering the field, to better understand the physics and the biology of TBI so to better select appropriate models to their research. We believe that the current models of TBI need refinement and that the first step toward high fidelity modeling is the understanding of the physics and the physical forces. We also believe that only those models should be used that mirrors the physical forces causing human TBI. Lastly, but most importantly, we hope that our effort will contribute to provide better care to victims of TBI in the form of specific diagnosis and treatments. In the age of “wearable devices” that can monitor a multitude of parameters connected by of “Internet of things,” and analyzed by using “big data” approaches, it is not too far fetched to see a completely novel ways coming “online” to model, diagnose, and treat TBI.

## Conflict of Interest Statement

The authors declare that the research was conducted in the absence of any commercial or financial relationships that could be construed as a potential conflict of interest.

